# The Present Development and Tendencies of the Dental Profession

**Published:** 1871-09

**Authors:** E. Blake


### ARTICLE II.

### *The Present Development and Tendencies of the Dental Profession.*

By Dr. E. Blake.

Extract of an address read before the Massachusetts Dental Society.

* * * * * It cannot have escaped the discernment of many among us, that our profession is in a transition state between an old and a new era. No one may be able to fix the date which marks the dividing line, but all are cognizant of the fact that the organization of dental societies —the institution of dental colleges—and the advent of dental literature—have wrought a change in our whole manner of thinking and feeling in relation to our calling. This change arises not merely from the fact that these institutions have enlarged the boundaries of our knowledge, or disseminated more widely and quickly all improvement in our methods of practice. It lies deeper, and results from causes more profound. It is because these have changed us from isolated individuals, working chiefly for personal ends, to a great fraternity of co-laborers, having common aims, interests, and working for the honor of our profession and the well-being of our race. These also open the way to the talented and ambitious, to prizes of title and honor, to wider usefulness and increased emoluments; thus stimulating exertion and securing progress. And through these we all become "Members one of another." Somewhat of the halo of glory that crowns the brow of the chiefest among us, becomes the valued heritage of the humblest in our ranks. Whatever fortune lifts one of our number to eminence, we all rejoice in his triumphs, and feel that he is still bound to us by the subtle ties of a similar discipline in like studies, labors and difficulties.

Before proceeding with our subject, let us for one moment consider the *nature* of this Profession of Dentistry, which we so prize and love, for which all these institutions exist, and in which are garnered all our hopes and aspirations for usefulness and success in life.

It has been common to speak of dentistry as a specialty of medicine. And not a few dentists have seemed to think that by hanging upon the outskirts of that profession they were obtaining a position of honor. But the dentist who is content with this definition and position of his profession, has a very inadequate ideal of the true dimesions, scope and dignity of his calling. The treatment of any particular organ constitutes a specialist in medicine. The aurist and oculist are specialists. And thus the treatment of the teeth and their incident diseases would constitute a specialist in medicine. But is this the whole of dentistry? Are there no other large departments of our profession in which Art is the presiding genius? and others again purely mechanical? It does not follow, because the occulist and surgeon are specialists in medicine, that, therefore, the manufacturers of artificial eyes and limbs are so too. In the mechanical department, the dentist is a moulder in sand, a worker in metal, in gypsum, and in porcelain. The manufacture of artificial dentures has nothing more in common with the theory or practice of medicine than has sculpture or painting. A knowledge of artistic and mechanical elements not needed by physicians or taught in their schools is here required. The artist must well understand the external expressions of anatomy, though he need not know its intimate structure. A like necessity rests upon the dentist in his artistic operations to restore the organs and forms of nature. But while this necessary obedience to the laws of art allies him to the artist, he must also understand the influence of his operations upon living tissues. And this knowledge, together with that involved in surgical operations still more nearly affecting vital functions, allies him to the physician. Thus these different elements are combined and blended in him As a mechanic he manipulates his materials, as an artist he decides their forms and eolors, as a specialist in medicine he fixes the nature and time and limits of all operations, as a dentist he does all. The mechanical and artistic elements are not less integral parts of dentistry, than are the surgical operations within the oral cavity. Yet we cannot conceive of them as within the province of medicine or medical study.

It would seem then that dentistry, including in that term all that is universally understood by it, cannot properly be claimed, indeed, that the bonndaries of dentistry are larger than those of medicine, because all medical knowledge may be useful, and a certain amount is indispensable to constitute a dentist a safe and efficient practitioner, while a physician may be ignorant of large departments of dentistry without impairing his professional position or usefulness. But still, in practice, considering that the sphere of our operations is more limited and that they are less vital to the health and lives of our patients, while that of the physician not only has a greater range and volume, but involves a mass of detail in physiological function and pathological condition, and of every substance and influence potent to control them, we must concede that the preponderence of science and dignity rests with the medical profession. I would detract nothing, but give all due honor to them. For let us always remember that in all that domain of our profession which we share with them we have a common heritage in the rich legacy of science accumulated through centuries of patient labor and research. Their masters are our masters. Their systems and methods are our guidss and monitors. The great names that speak with authority to them are our high priests as well. Those presumptuous individuals sometimes found among us, who butt their brainless heads against the solid masonry of fixed medical knowledge, deserve neither patience or consideration. Some of this class—neophites in science—who know nothing yet as they ought to know—are ever obtruding their fantastic theories as veritable discoveries which are to revolutionize the whole art of dentistry and the whole science of medicine. Let not pigmies attempt the task of titans. Blind like Samson, yet without his strength, they cannot pull down the temple of science upon our heads. A full course of medical study would correct these follies. But though desirable, it is not indispensable to entrance into our profession. And certainly a medical education is by no mean equivalent to a dental education—*for dentists.* We all know that some who came to us from that profession are in no wise superior operators to others whose first essay in life was made in other callings. Above all, we must not allow our profession to become, in any degree, a mere appendage to that of medicine. In truth our family pedigree runs back to different roots. If medicine is our father, Art is our mother. If we are brothers to the physician, our relationship is very near to the painter, the sculptor and the jeweller. If our profession is less purely intellectual than Divinity or Law, less comprehensive and cumulative of detail than Medicine, less æsthetic than Art, and taxes physical endurance less than mechanical labor, it yet makes larger demands upon the facilities necessary to each, and developes the whole man in a more harmonious manner than either of them.

Cherishing then these large and catholic views of the nobleness of our profession, recognizing it as a new one born of the exigencies of modern life, but yet the inheritor of sciences and arts long cultivated, we may well exert our most earnest efforts for its improvement and elevation, and the proper recognition of its claims. Drawing its life from such rich sources, freighted with so many blessings to humanity, it can have no other future than one of constantly expanding usefulness. And since it is our lot to stand in the very focus of this transition period, moulding and influencing its destinies more than the men of the past had power, or those of the future will have opportunity to do, let us make sure that it becomas a distinct entity—*sui generis*—taking its own form—living its own life, grand and noble—apart from all others.

Dentistry has obtained a higher development and a wider diffusion within the United States than in any other country. This arises from two causes: First, the greater frailty and earlier loss of teeth among our people; and, Secondly, the more general diffusion of wealth and intelligence, which enables a much larger proportion of the people to call to their aid the services of the dentist. Finding itself thus conditioned, the profession, which embraces within its ranks many scientific and ingenious minds, has not been slow to push its conquests, and has wrought out for itself its present high position.

* * * * * Of the present tendencies of our profession it may be remarked that the grand central influence is one of progress and development in all directions; financially, intellectually, morally. Not only is the average dentist of to-day better remunerated than at any former time, but a more generous culture, a more thorough professional training, and a higher standard of character is exacted and maintained by the profession. Voluntary associations having these ends in view, are general throughout the country. Our colleges and literature sustain and help forward the good work, and the great body of the profession are brought into more intimate relations with each other and inspired with better sentiments than ever before.

Among the special tendencies of our time, voluntary associations for the promotion of professional objects stand pre-eminent. Dental societies were first to take the field as an educating power. They have generally acted in a liberal spirit, extending their arms to receive all worthy applicants, with or without diplomas—if only coming with sufficient evidence of professional standing, with a clean record, and in a fraternal spirit. And whatever reputation and position any one may possess, he loses nothing, but gains much, by such associations with his professional brethren. But those societies, to reach their highest usefulness, must act on broad, liberal and permanent principles. The ambitions of men, the selfishness of cliques, the narrowness of parties, should find no place in them. The elevation and improvement of the profession, and the protection of the public from quackery, are the true objects to be secured. And could any society be turned aside from these worthy objects to the advancment of low aims, the certain and deserved result would be, loss of public confidence, rivalries and jealousies among the members, and the final disintegration and ruin of the society. While it is doubtless true that these elements of danger and discord exist in some degree in all our societies, I am glad to believe that the sound portion, loyal to truth and duty, will generally assert its rightful supremacy. The low arts which too often find their place in trade, make the general public suspicious of all pretensions to strictly honest dealing. Though imposture may endure for a time, yet at last all gaseous baubles will surely collapse, and all imposing shams be turned inside out. Not only must the aims and acts of our dental societies be guided by high and honorable principles, but this fact must be patent to all the world. It must be seen and felt that we exist no less for the public safety than for our own advantage. The time will come—there is no reason it should not be so now—when all regular dentists within fixed geographical limits will be members of one society, as physicians now are. This right of being a member by reason of being a dentist may be subject to certain qualifications, as with them. But it must be placed wholly above the exigencies of the society government, or of rival cliques. Any other course is demoralizing to the members, and must finally prove disastrous to the reputation and success of the society. There can be no ostracism in the world of science, any more than in the world of letters. Social, class, or personal antipathies have no right to obtrude their hateful presence within these charmed circles. Each left free to show his quality and power, he stands or falls by his merits.

The very first condition of success is harmony, and the respectful concession of all its rights to all its members. Nothing so fatally destroys all hope of usefulness as discord. From the ancient Psalmist who exclaimed, "Behold how good and how pleasant it is for brethren to dwell together in unity," to the utterance of Him who spake as never man spake, "Blessed are the peacemakers, for they shall be called the children of God," the benedictions of God and man have ever been upon the lovers of their race and the promotors of peace. If ambition has sown our earth with dragon's teeth, when it has fired the bosom of some great military chieftain, evils as great, if not so malign, have been its fruit when exhibited in other fields. Ambition, said to be the last infirmity of noble minds, is often the first infirmity of minds ignoble, and its evil influence is felt in every organization where honors are to be seized, or position and influence are to be won. Even societies like our own, whose legitimate sphere is the cultivation of scientific knowledge for the purpose of alleviating human suffering, have not been exempt from its baleful power. In exercising so fell a spirit, each should do something to save our societies from distrust and approbium.

The tendency to follow specialties in dentistry is already well marked. In several of our large cities there are establishments exclusively for the extraction of teeth, and the separation of the mechanical and artistic, from the surgical departments has long been proceeding with accelerating force. Though the men and methods effecting these results thus far, may be condemned, it will be admitted that the division is itself neither unreasonable or undesirable, and where the population is sufficiently dense, is likely to be pefected in the future. The hand, used to the coarser labor of the laboratory, is not fit, the next minute, to touch the delicate cheek of beauty, or to perform the dexterous manipulations that new-crown a tooth with gold. Even where these branches are carried on in the same office, under the same supervision, both kinds of work are seldom performed by the same man. They naturally cleave asunder and gather into fitting hands. It is not necessary to decide which is of higher grade. It is enough that they are diverse and incongruous. In a great metropolis, why should not extracting constitute one speciality, the treatment of irregularities another, the manufacture of artificial dentures another, and filling teeth still another, which latter of course would employ the great body of the profession. What valid objection could be made to this, provided these specialties were followed by regular members of the profession, and did not, as now they sometimes do, fall into the hands of men, who, to say the least, are very loosely connected with it. All this is in process of development, whether we may view it with complacency or not. And let us hope it will subserve equally well the interests of the public and of the profession, since all division of study or labor, which does not dwarf the mind, concentrates power and promotes perfection.

Another tendency of the present time, one of the strongest as well as one of the best, is towards the conservation of the natural teeth as opposed to the substitution of artificial dentures. Artificial teeth at best are merely substitutes, only tolerated because they are our last resort. Only imperative necessity reconciles any one to the loss of any organ or part of the body. My living tooth like my arm is part of myself. When it is lost, a part of my own proper person is consigned to the grave. But an artificial denture is only so much pyroxiline, rubber, porcelain, gold and platinum—so much metal, gum and stone. Like filthy lucre, "'Twas his, 'tis mine, and may be a to slave thousands." Like a garment, if it fits and suits, one may wear it; if not, he throws it aside without compunction. It is true, it is a work of art to form from the plastic elements a denture, conforming to the general anatomy, harmonizing with the complexion and features, performing the offices of use also— articulation and mastication; all this and more—doing *its* part in giving expression to the character of the heart and soul—this were indeed a work of high art. But no art or science or skill ever did or ever can give back the pefection which nature —the grandest of artists, and the most loving of mothers— bestows upon all her children. No anathema is too severe for him who mars her work, and for gain, recklessly sacrifices the natural teeth—no praise too high for him who saves them. With what conscientious faithfulness then, ought we to labor for those who confide them to our care! Forty years ago, the insertion of artificial dentures constituted the chief part of dentistry. To-day its proportion is much less, showing the great relative progress of conservative dentistry.

Other professions have had centuries of development and growth. But as Minerva leaped from the brain of Jupiter in the full panoply of war, so Dentistry came forth from the brain of the nineteenth century, in full vigor and maturity, to meet the need of an advancing civilization. Eminent individual practitioners there have been, but no dental profession, known, and respected as such until our own day. Yet, young as it is, the charge sometimes made that it is the peculiar home of quackery, is unfounded. The sun has dark spots—barnacles cling to the best of ships—and it is said that Satan can put on the shining garb of an angel of light. Charlatans and pretenders abound everywhere—in law—in medicine; wolves in sheeps' clothing sometimes invade the sacred desk. Is it any wonder, then, that many camp-followers of fortune should seek our cultivated fields, promising as it would seem to them, such easy spoils? Like the frogs and lice of Egypt, they invade the hovel and palace alike—like its locusts, they eat up every *green thing*. Many people of wealth, and some of high mental culture and social position, run after the most arrant impostors in dentistry and medicine. It is one of the profoundest and saddest mysteries of human nature that it will go far and seek long for folly, when wisdom is right within its grasp. Yet, spite of this general lack of discrimination and appreciation in the public, by the healthy vitality developed within itself, effete and gangreneous methods and members are sloughed off—learning, skill and judgement are recognized, and rapid progres is made by the profession.

Our profession has by no means yet reached its maximum ratio to the population. There are still great masses of people whose mouths are full of pain and rottenness and ruin, with offensive breath, and who are utterly incapable of masticating their food. Under these conditions digestion is never properly begun, and dyspepsia is an ever-present demon. The field is large. Let us not consider our profession too full till all this multitude is reached and purified. The highest art should always take precedence where it can be remunerated; but where it cannot, do not shut men out from health and blessing which cheaper methods and material can supply. If gold is unattainable, better amalgam than decay and pain. Better inartistic teeth of disagreeable whiteness and mechanical regularity on clumsy rubber plates, than to have the mouth cumbered with painful and offensive old roots. Though showing a common honesty and a common science, of necessity, dentists must differ in style and method and grade to meet the needs of the different strata of society. There is room for all. Let us all mount as high as we can upon the ladder of success, but let us not shake down, from their low perch, those still trembling and struggling with fate upon the lower rounds.

"Men die, but institutions live." And we may hope that, long after we have passed away, this Society will still live and still remain an honor to the profession and useful to the world. It may be, the time will come when man, having filled the world with machinery and perfected his domestic animals, will find time to improve himself. If that golden era ever does come, we may be sure that dentists will be in demand. Not, as now, to remedy defects, but in the higher office, to prevent them. Incipient mothers will come to him for advice. Children will be trained under his eye and with his counsel. Chemistry, hygiene and art will unite their mystic forces so secure a sound body—the only possible dwelling-place of a sound mind.

It may well be a matter of pride to us that the American dentist leads the world, in an art that, more nearly than any other, marks the progress of a people in wealth, intelligence and refinement. Where a skilful and honest dentist is not appreciated, there the people are ignorant or thriftless, or coarse or vicious, or all combined. Knowledge and cultivation prompt to constant watchfulness over the agencies of evil that are ever marshalled against these tenements of clay—so "fearfully and wonderfully made." Health and vigor are so dependent upon good digestion, and this again upon good teeth, that the loss of these opens a wide avenue to the invasion of disease and death. "Cleanliness is next to godliness." A clean, pure, healthy body, helps mightily to saintliness of soul. The mouth is the vestibule of the body. If that is not kept swept and garnished, there is no hope of finding purity within.

In this transition period of our profession, if all is not gold that glitters, and some hold forth a science falsely so called, and others temporarily ride the wave with honors to which they have no claim, we must consider it the evil of the situation and wait patiently for the calmer day when the men and work of to-day will be weighed in standard scales, and be stamped with their true and absolute value and character. But with all our deficiencies and errors, let us hope that the present works and records of American dentistry will contribute some of the brightest evidencies of the achievements of our era in those graceful arts which adorn and dignify human life.

As members of a profession it is not ours to accumulate large wealth or to gather civil honors. The only ambition we should cherish must find its accomplishment in ideal excellence. Next to our religion and our domestic ties, devotion to professional duty should be the controlling sentiment of our lives. Let us idealize our profession; let us devote ourselves to it as a science; let us love it as an art.

Let us see beauty in our handiwork—in the glitter of jewelled teeth—in the naturalness and harmony and artistic blending of all excellences in our substitutes for the pearly organs of nature. Let us find pleasure in the good we do—in the prevention of pain—in the comfort and enjoyment we render possible—in the restoration of beauty to the deformed—in the healthful vigor we increase and prolong. Let us not expect or seek full remuneration in money for all this weary labor of hand and eye and brain. So much money for so much work would be poor and unsatisfying recompense to men imbued with love of science and art. But rather let our reward be in the satisfaction that comes from high attainment—from the appreciation and gratitude of patients who realize the benefits they receive—from the respect of professional brethren—from anticipation of the good name and fair fame we shall leave behind us.

Many of us, alas! will not live to realize the full fruition of our work in large business and fees. But not one—who loves his profession—who aims high—who marches toward pefection with unflagging step—shall miss the reward that comes from consciousness of high endeavor and duty done. This does not wait for external recognition, but is a perpetual fountain of joy in each man's inner life. With so many incentives to faithful labor and high achievement, giving our lives to science—to art—to duty—to man—to God—our pathway is onward and upward.

